# Evaluation and Application of the RD_50_ for Determining Acceptable Exposure Levels of Airborne Sensory Irritants for the General Public

**DOI:** 10.1289/ehp.9848

**Published:** 2007-08-07

**Authors:** Yu Kuwabara, George V. Alexeeff, Rachel Broadwin, Andrew G. Salmon

**Affiliations:** Office of Environmental Health Hazard Assessment, California Environmental Protection Agency, Oakland, California, USA

**Keywords:** Alarie test, exposure levels, LOAEL, RD_50_, REL, sensory irritation, TLV

## Abstract

**Background:**

The RD_50_ (exposure concentration producing a 50% respiratory rate decrease) test evaluates airborne chemicals for sensory irritation and has become an American Society for Testing and Materials (ASTM) standard method. Past studies reported good correlations (*R*^2^) between RD_50_s and the occupational exposure limits, particularly threshold limit values (TLVs).

**Objective:**

The main purpose of this study was to examine the relationship between RD_50_s and human sensory irritation responses in a quantitative manner, particularly for chemicals that produce burning sensation of the eyes, nose, or throat, based on lowest observed adverse effect levels (LOAELs) reported for human subjects.

**Methods:**

We compared RD_50_s with LOAELs and acute reference exposure levels (RELs). RELs, developed by the California Environmental Protection Agency’s Office of Environmental Health Hazard Assessment, represent a level at which no adverse effects are anticipated after exposure. We collected RD_50_s from the published literature and evaluated them for consistency with ASTM procedures. We identified LOAELs for human irritation and found 25 chemicals with a corresponding RD_50_ in mice.

**Discussion:**

We found the relationship between RD_50_s and LOAELs as log RD_50_ = 1.16 (log LOAEL) + 0.77 with an *R*^2^ value of 0.80. This strong correlation supports the use of the RD_50_ in establishing exposure limits for the public. We further identified 16 chemical irritants with both RD_50_s and corresponding acute RELs, and calculated the relationship as log RD_50_ = 0.71 (log REL) + 2.55 with an *R*^2^ value of 0.71. This relationship could be used to identify health protective values for the public to prevent respiratory or sensory irritation.

**Conclusion:**

Consequently, we believe that the RD_50_ has benefits for use in setting protective levels for the health of both workers and the general population.

Although airborne chemicals can cause a number of harmful effects, the most common effect is sensory irritation (De Ceaurriz et al. 1981). Exposure to a sensory irritant may stimulate the trigeminal nerve endings and laryngeal receptors, eliciting any one or a combination of the following symptoms: burning sensation of the eyes, nose, or throat, as well as coughing sensations (Alarie et al. 2000). Sensory irritation is also the most common end point for occupational exposure levels (OELs). For one specific OEL measure, threshold limit values (TLVs) [developed by the American Conference of Governmental Industrial Hygienists (ACGIH 2006)] are calculated based on sensory or pulmonary irritation for > 50% of the compounds. Kane et al. (1979) reported that approximately two-thirds of the compounds for which they found a TLV acted as sensory irritants. A qualitative evaluation of sensory irritants indicated that sensory irritation responses in the mouse are predictive of responses in humans (Alarie 1973a).

In 1966, Alarie initially proposed the use of an animal test to evaluate the potency of airborne sensory irritants. The bioassay uses male Swiss-Webster mice to measure decreases in respiratory frequency resulting from exposure to a geometric series of concentrations of airborne irritants (Alarie 1966). The concentration inducing a 50% decrease in respiratory frequency is termed the RD_50_. From these measured RD_50_s, Alarie (1981b) ranked irritant potencies and found a good correlation (*R*^2^) between RD_50_s and TLVs. The Alarie test evolved over the years and was adopted in 1984 as a standard test by the American Society for Testing and Materials (ASTM 2004). The “RD_50_ test” or the “Standard Test Method for Estimating Sensory Irritancy of Airborne Chemicals” (ASTM 2004) quantitatively measures irritancy as indicated by the reflex inhibition of respiration in mice exposed to sensory irritants. For the test, four mice are first acclimatized to the chamber and are then simultaneously exposed to the airborne chemical. A sufficient number of groups are exposed to a geometric series of concentrations so that a concentration–response curve can be constructed from the analysis. The mice are placed in a body plethysmograph attached to an exposure chamber so that only the head is exposed to the test material. The plethysmographs are connected to pressure transducers, which sense changes created by inspiration and expiration. The amplified signals are transmitted to a polygraph recorder. The concentration of airborne irritant that produces an RD_50_ is determined from the concentration–response curve constructed from the various data points obtained with a series of concentrations.

Sensory irritation is a reflex reaction from stimulation of the trigeminal or laryngeal nerve endings (Boylstein et al. 1996). The sensory irritant response is mediated through binding to the trigeminal nerve receptors and appears to follow Michaelis-Menten receptor kinetics. Although the RD_50_ concentration has been described as “intolerable” to humans, as indicated in the ASTM standard, “the test method will detect irritation effects at concentrations far below those at which pathological changes are observed” (Alarie 2000; ASTM 2004). Further, as demonstrated by Barrow et al. (1986), pathologically detectable responses are expected only after prolonged repeated exposure.

RD_50_s are a basis, at least partially, for a number of OELs by ACGIH (ACGIH 2006). The calculation methodology is based on Kane et al. (1979), who evaluated data from 11 sensory irritants and concluded that a level one-hundredth of the RD_50_ would produce “minimal or no sensory irritation” in humans. The current suggestion of setting OELs at 0.03 RD_50_ comes from Alarie (1981a, 1981b), because 0.03 RD_50_ is halfway between 0.1 RD_50_ and 0.01 RD_50_ on a logarithmic scale. Alarie (1981a) reported a strong correlation (*R*^2^ = 0.89) between 0.03 RD_50_ and OELs for the 26 chemicals tested. Subsequently, both analyses, one using 41 chemicals (Alarie and Luo 1986) and most recently another using 89 chemicals (Schaper 1993), resulted in a lower but still strong correlation (*R*^2^ = 0.78). Although most of the applications of the RD_50_ have focused on OELs, Nielsen et al. (1995) found that protection against indoor sensory irritation effects could be achieved at a level of 0.025–0.25 of the OEL. Multiple studies show strong correlations between RD_50_s and OELs, supporting the continued use of the Alarie test for establishing OELs (Kane et al. 1979, 1980; Schaper 1993).

In this study we examined the relationship between RD_50_s and human sensory irritation responses in a quantitative manner, particularly for chemicals that produce burning sensation of the eyes, nose, or throat, based on lowest observed adverse effect levels (LOAELs) reported for human subjects. We also analyzed the relationship between RD_50_s and OELs for identified human sensory irritants. Finally, we evaluated the relationship between RD_50_s and acute reference exposure levels (RELs) developed to protect the public (Collins et al. 2004). RELs are defined as “[t]he concentration level at or below which no adverse health effects are anticipated for a specified exposure duration [1 hr for the acute RELs]. … RELs are based on the most sensitive, relevant, adverse health effect reported in the medical and toxicological literature.” A strong correlation between RD_50_s and LOAELs, TLVs, and acute RELs will support the use of RD_50_s in establishing guidance levels to protect the public from sensory irritants.

## Methods

### LOAELs versus RD_50_s

Using published toxicologic studies of human subjects exposed to sensory irritants, we identified human LOAELs. Criteria for selecting human LOAELs required that the studies describe mild irritating effects (Alexeeff et al. 2002) resulting from acute inhalation exposure. Published human studies on hazardous air pollutants (HAPs) served as the primary sources of information for these chemicals (Alexeeff et al. 2002). We searched PubMed (National Library of Medicine; http://www.ncbi.nlm.nih.gov/sites/entrez), Biosis (www.biosis.org/), Current Contents (http://scientific.thomson.com/products/ccc/), Toxline (National Library of Medicine; http://toxnet.nlm.nih.gov/cgi-bin/sis/htmlgen?TOXLINE), SciFinder Scholar (Chemical Abstracts Service; http://www.cas.org/support/scifi/sfsolutions/index.html), Oldmedline (http://www.nlm.nih.gov/databases/databases_oldmedline.html), Web of Science (http://scientific.thomson.com/products/wos), and Environmental Sciences and Pollution Management Databases (Cambridge Scientific Abstracts; http://www.csa.com/factsheets/envclust-set-c.php) to identify toxicologic studies published between 1970 and 2005 for all 189 HAPs. Search terms included the chemical name, the type of LOAEL effects (e.g., irritation), route of exposure (inhalation), and exposure duration (acute). We also conducted online searches for additional non-HAP chemicals with an identified RD_50_. Further, we conducted manual searches from secondary sources through 2005. Five criteria were developed for inclusion of a study in the analysis: *a*) peer-reviewed and published, well-conducted industry-sponsored studies or doctoral dissertations; *b*) inhalation exposure; *c*) discrete acute exposure; *d*) available LOAEL for a mild adverse health effect; and *e*) the original research. For each human study analyzed, information about the chemical, exposure time, end-point category (eye and/or respiratory irritation), and LOAELs were recorded. If multiple mild responses were reported at various dose levels for the same chemical and exposure time, then the lowest adverse effect level was considered the LOAEL.

For RD_50_s, we first reviewed references identified from the database developed by Schaper (1993). We identified additional studies from Alarie et al. (2000). We also searched the scientific literature during the period 1992–2005 to identify newer published studies containing RD_50_s. For each identified study, we recorded information on the chemical, exposure time, species, and RD_50_. We reviewed the methodology used to attain each RD_50_ for consistency with current ASTM methods (ASTM 2004); for this reason, we included studies with mice, but excluded studies with rats in this analysis.

In cases where both RD_50_s and human LOAELs were available for the same chemical, we log transformed and fit the data with a linear relationship using Microsoft Office Excel 2003 (Microsoft, Redmond, WA) and SAS version 9.1 (SAS Institute Inc., Cary, NC) for Windows. This procedure was similar to previous RD_50_ comparisons (e.g., Alarie 1981b). When we found multiple LOAELs or RD_50_s for a single chemical, we considered each reported value in the analysis. Sensitivity analyses were conducted by evaluating the correlation generated from the regression of LOAELs with RD_50_ value data sets, which varied by exposure time, or strain tested. We also conducted subanalyses using upper and lower respiratory tract effects.

### RELs versus RD_50_s

As reported by Collins et al. (2004), the California Environmental Protection Agency (EPA) has developed 51 acute inhalation RELs. We evaluateds these RELs to identify those based on eye or respiratory irritation end points in humans, and compared with RD_50_s. Using Microsoft Office Excel 2003 (Microsoft) and SAS version 9.1 (SAS) for Windows, we log transformed and fit the data with a linear relationship.

### TLVs versus RD_50_s

For all RD_50_s used in the above analyses, we identified TLVs from ACGIH (2006). The TLVs included time-weighted averages, short-term exposure limits and ceilings. If the documentation reported more than one TLV value, we used the lowest, more protective value. A third comparison between RD_50_s and TLVs of identified human irritants, based on identification of a human LOAEL for irritation, was conducted using log-transformed data, fit with a linear relationship, and analyzed with Microsoft Office Excel 2003 (Microsoft) and SAS version 9.1 (SAS) for Windows.

## Results

### LOAELs versus RD_50_s

From our search, we identified 25 chemicals with 72 human acute irritation LOAELs from 49 studies (Table 1). The adverse effects, exposure times, and information reflecting the quality of the study (e.g., placebo-control, blinding, subject selection, subject characteristics, exposure design, and data reporting) are indicated in Table 1. For the 25 chemicals identified, 63 RD_50_s were found in mice (Table 2). The RD_50_s were based on seven mouse strains and exposure times ranging from 5 to 180 min.

**Figure 1:**
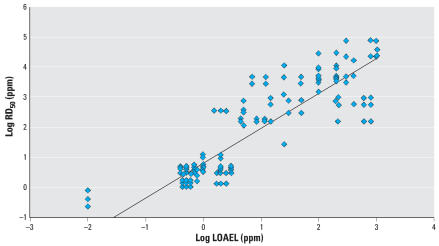
Linear least-squares regression analysis for log RD_50_ (for all mouse strains) vs. log LOAEL (human irritation end points) for 25 compounds, using 195 data points. Log RD_50_ = 1.16(log LOAEL) + 0.77; *R* ^2^ = 0.80.

Figure 1 shows the correlation between RD_50_s and LOAELs for all RD_50_s identified in all strains of mice for the 25 chemicals, allowing for 198 comparisons. There is a strong overall correlation (*R*^2^ = 0.80) between RD_50_s and human irritation LOAELs. When we conducted the analysis for Swiss-Webster mice only (Table 3), we were able to include 75 data points for 19 compounds, and the correlation decreased slightly (*R*^2^ = 0.74). When we evaluated only the data for non–Swiss-Webster mice (Table 3), there was little change in the correlation (*R*^2^ = 0.83). We conducted several sub-analyses to consider the influence of the RD_50_ study exposure duration. As indicated in Table 3 there was little influence on the *R*^2^. Thus, according to this analysis, the strain of mouse tested does not appear to affect this evaluation substantially. The equations do not change significantly, and the correlation is still significant for all analyses, validating the inclusion criteria used. As indicated in Table 3, we also considered several subanalyses to address the influence of the human LOAEL variability. Specifically, we considered the issue of LOAEL sensitivity, the type of irritation end point, study quality, and the duration of exposure for the human LOAEL. The only significant effect on the correlation was observed when considering human irritation end points of the lower respiratory tract; the poor *R*^2^ appears to be attributed partly to the few number of data points (29) in the analysis.

### RELs versus RD_50_s

**Figure 2:**
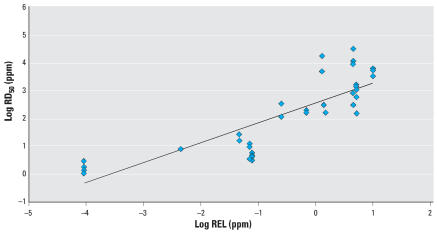
Linear least-squares regression analysis for log RD_50_ (mice) vs. log REL (set by OEHHA for airborne toxicants) for 16 compounds. Log RD_50_ = 0.71(log REL) + 2.55; *R* ^2^ = 0.71.

From the 51 California acute RELs, we identified 16 that had irritation as their end point and a corresponding RD_50_. Figure 2 indicates a good correlation (*R*^2^ = 0.71) between RD_50_s and RELs for 16 chemicals with 37 comparisons.

### TLVs versus RD_50_s

**Figure 3:**
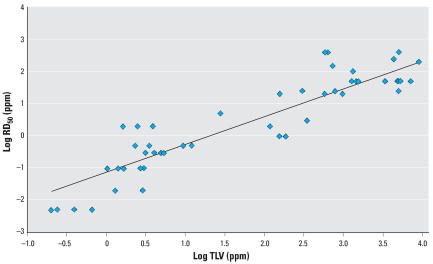
Linear least-squares regression analysis for log RD_50_ (male mice) vs. log TLV for 24 compounds (no TLV for *n*-pentanol). Log RD_50_ = 0.86(log TLV) – 1.13; *R*^2^ = 0.86.

For the compounds identified with RD_50_s and LOAELs, 24 had a corresponding TLV. Figure 3 shows the correlation of TLVs to RD_50_s with an *R*^2^ value of 0.81. Thus, when focusing specifically on human irritants, the relationship between the TLV and RD_50_ remains strong.

## Conclusions

The focus of this paper is on the applicability of RD_50_s for human health risk assessment. Exposure guidelines to protect workers and the public often focus on mild irritating signs or symptoms. For example, > 50% of the TLVs and > 60% of the California acute RELs based their end points on irritation (Collins et al. 2004). However, human studies from which to develop acute exposure guidance are not available for many of the hundreds of substances of concern, and therefore reliance on animal studies is necessary. The RD_50_ test method is appealing because it generates data rapidly, requires minimal animal use, is low in cost, and is validated, calibrated, and standardized. The method was computerized, adding to the reproducibility of the results (Alarie 1998, 2000; Vijayaraghavan et al. 1994). The availability of RD_50_s in male mice for 89 chemicals (Schaper 1993), and their correlation with OELs suggests potential applicability to air exposure guidelines for the public. The result of this analysis quantitatively supports the applicability of RD_50_s in setting exposure guidelines for the public and workers.

We found a strong correlation between RD_50_s and human LOAELs, TLVs, and California RELs. Focusing on human studies where the subjects developed eye or respiratory irritation responses, we observed a strong correlation (*R*^2^ = 0.80) between RD_50_s and LOAELs for 25 chemicals with irritating effects. The correlation remained close to 0.8 after conducting various subanalyses, indicating that the strains of mice or the RD_50_ exposure time does not substantially affect the correlation. Previously, Nielsen et al. (1995) proposed an indoor air guideline for the public between 0.025 and 0.25 times the OEL, similar to 0.0008 and 0.008 times the RD_50_. In our analysis, the RD_50_ to REL correlation can be expressed as REL = 0.00026 × RD_50_^1.4^. Derived as follows:


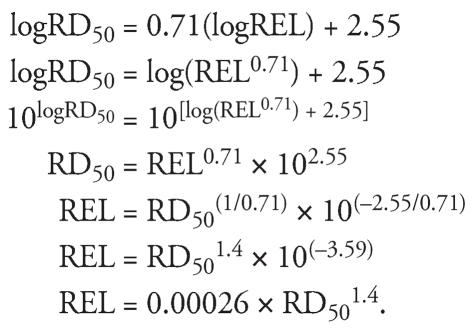


Exposure times in the human studies varied from 1 to 480 min, and a subanalysis looking specifically at the effect of the duration of exposure made no significant change to the correlation. Further, subanalyses using LOAELs more closely associated with either upper respiratory or lower respiratory effects did not make a significant change to the correlations. Although the variability in the response rate, interindividual sensitivity, and differences in human study design, as described in Table 1, would be expected to have reduced the correlation with the RD_50_, specific factors were not identified in our subanalyses. Thus, we conclude that the irritating symptoms in humans correlate well with the RD_50_s of animals irrespective of the specific acute exposure duration. These results not only support the use of the RD_50_ in setting guidelines for acutely irritating compounds, but also suggest that a concentration–time extrapolation for these effects appears unwarranted. This is consistent with the finding by Shusterman et al. (2006) that the human response to sensory irritants reached a plateau rapidly. Thus, the response appears to be influenced to a greater extent by the exposure concentration rather than the exposure time over the period of observation for most animal and human experiments considered in the present analysis, and over the periods of concern for the TLVs (15 min to 8 hr) and acute RELs (1 hr).

The results of this analysis are subject to several limitations. First, the number of available human studies limits the LOAEL data, and it is unlikely that human data will significantly increase in the future. The number of comparisons could increase as the numbers of RD_50_s increase for chemicals with human data. However, considering the robustness of the subanalyses, and the historical correlation of the RD_50_ to the TLV, a significant change in the RD_50_ to LOAEL correlation is unlikely after adding other sensory irritants in the analysis. Finally, we address issues raised by Bos et al. (1992, 2002, 2003).

First, Bos et al. (2003) claimed that the RD_50_–OEL correlation is expected because most OELs are based on animal data. Although many OELs are based on animal data, many are based on human data as well. Of the 24 substances we evaluated in our RD_50_–OEL correlation, the OEL for only one compound, *n*-pentyl acetate, relied on the RD_50_ for its derivation, which was based solely on animal data. The strong correlation between RD_50_s and human LOAELs also addresses this concern.

Second, Bos et al. (2002) reported the RD_50_s did not correlate well with histopathologic changes in the respiratory tract or with corrosivity, and therefore RD_50_s were inappropriate to evaluate respiratory tract irritation. However, the stated purpose of the ASTM standard is to evaluate sensory irritation potential, not histopathology or corrosivity. In our comparison of the RD_50_s with human irritation LOAELs, the correlation was strong with the inclusion of respiratory tract irritation end points in the analysis. Further, the risk assessment framework for occupational and public exposure levels addresses the concerns regarding the potential for other, more severe effects. In cases where other health effects occur at or below levels producing sensory irritation, exposure guidelines use the more sensitive adverse effect.

Third, Bos et al. (1992) raised concerns regarding the inconsistency of RD_50_s among strains and species. Although RD_50_s have been generated for various strains and species with varying test procedures, adhering to the ASTM standard method addresses this concern. Limiting the RD_50_ test to those conducted in mice, or Swiss-Webster mice, and limiting the exposure time keeps the test to a more standardized method, although intrastrain variability was not a cause for concern in our subanalyses. Finally, we addressed the concern regarding time–concentration response curves (Bos et al. 1992), with separate subanalyses based on exposure time. These analyses show that time did not appear to be a factor in our analyses. Our presumption is that if the study adheres adequately to the ASTM standard method, experimental exposure time plays a minor role. It is also worth pointing out that all of the figures comparing RD_50_s to LOAELs, RELs, and TLVs are plotted on a log–log plot because of the wide range of values. Because of the nature of log–log plots, the correlation is higher compared with the same correlation using a nonlogarithmic scale.

The applicability of the RD_50_ test to human health protection has been demonstrated in several analyses, but extrapolation of the test results to the general public would be improved with greater focus on the tail of the dose–response curve, to ensure protection of sensitive subpopulations. One solution would be for RD_50_ studies to report sufficient information to calculate a benchmark dose (BMD) value, and not focus solely on the specific RD_50_ value. A standardized BMD value could be calculated at the tail of the distribution, taking into account the slope of the dose–response curve. Alternatively, the test procedure could be refined to identify the “just detectable effect level,” which is approximately a 12% decrease in the respiratory rate (Alarie 1998). Although some work has been done in this area (Boylstein et al. 1996), additional information is needed to better understand the tail of the dose–response curve and to address any concerns for spurious results from low exposure concentrations. The reported just detectable effect level of 12% appears to be close to the no observed effect level of the procedure. Use of this response rate in risk assessment is consistent with the recommendation by the U.S. EPA (2007) that the BMD for a continuous response may be set on statistical criteria of distinguishability from the control value, as well as on grounds of anticipated biological significance. A major benefit of focusing on the just detectable effect level would be to reduce potential animal suffering, and possibly animal usage.

In conclusion, the RD_50_ test is a good starting point for setting exposure standards for acute airborne irritants. As noted by Alarie et al. (2000), the TLV may need to be < 0.03 RD_50_ to prevent other toxic effects. Consequently, the literature should be adequately evaluated to determine that sensory irritation is likely the most sensitive adverse effect. The application of RD_50_s appears most useful when qualitative data are available indicating sensory irritation as the most sensitive adverse effect, but quantitative human data are lacking. The RD_50_ has proven its usefulness with the ability to appropriately rank the potency of airborne chemicals as sensory irritants and help establish exposure limits. A strong correlation between RD_50_s and LOAELs provides further support for using RD_50_s in determining guidance levels to protect the general public from sensory irritants.

## Figures and Tables

**Table 1:** LOAELs for human sensory irritation for each study found in the literature.

Compound	LOAEL (ppm)	Time (min)	No. of subjects	% Response^a^	End point^b^	Reference
Acetaldehyde	7	5	27	0	Eye irritation	Stephens et al. 1961
	12	4	9	Average^c^	Bronchial hyperresponsiveness (L)	Myou et al. 1994^d^
	50	15	12	Majority	Eye irritation	Silverman et al. 1946
Acetone	300	3–5	10	Majority	Eye irritation	Nelson et al. 1943
	800	20	27	Average	Eye and weak nasal irritation	Dalton et al. 1997^d^
	990	240	16	100	Eye, mouth, and throat irritation	Seeber et al. 1992
	1,000	450	4	75	Eye and throat irritation	Stewart et al.1975
Acrolein	0.44	NG	10	NG	Conjuctival and nasal irritation	Plotnikova 1960
	0.5	5	36	20	Eye irritation	Stephens et al. 1961
	0.6	5	16	Average	Eye and nasal irritation	Hine et al. 1961
Allyl alcohol	0.78	5	6	Average	Slight nasal irritation	Dunlap et al. 1958
Ammonia	5	180	12	100	Eye irritation	Sundblad et al. 2004^d^
	30	10	5	40	Eye and nasal irritation	MacEwen and Vernot 1972
	50	30	16	44	Eye and throat irritation	Verberk 1977
*n*-Butyl acetate	200	3–5	10	Majority	Throat irritation	Nelson et al. 1943
*n*-Butanol	25	3–5	10	Majority	Eye, nasal, and throat irritation	Nelson et al. 1943
Chlorine	0.95	240	8	Average	Forced vital capacity decrease (L)	Rotman et al. 1983^d^
	1	60	5	Average	FEV_1_ decrease (L)	D’Alessandro et al. 1996^d^
	1	480	29	100	FEV_1_ decrease (L)	Anglen 1981^d^
	1	120	29	100	Urge to cough	Anglen 1981^d^
	1	60	29	100	Throat irritation	Anglen 1981^d^
	2	60	8	100	Urge to cough	Anglen 1981^d^
	2	240	8	100	Forced vital capacity decrease (L)	Anglen 1981^d^
	2	120	8	75	Throat irritation	Joosting and Verberk 1975
	2	60	8	25	Nasal irritation	Joosting and Verberk 1975
	2	30	8	38	Nasal and throat irritation	Joosting and Verberk 1975
Ethylacetate	400	3–5	10	Majority	Nasal and throat irritation	Nelson et al. 1943
	402	240	16	Average	Eye, nasal, and throat irritation	Seeber et al. 1992
Formaldehyde	0.4	120	20	Average	Rhinitis	Pazdrak et al. 1993^d^
	0.5	120	20	100	Nasal irritation	Krakowiak et al. 1998^d^
	0.69	480	109	Average	Eye irritation	Horvath et al. 1988
	1	120	16	44	Conjunctival irritation	Anderson and Molhave 1983
	1	6	27	100	Eye irritation	Bender et al. 1983
	1	5	75	8	Eye irritation	Stephens et al. 1961
	1	1.5	48	Average	Nasal irritation	Weber-Tschopp et al. 1977
	1	90	18	84	Eye, nasal, and throat irritation	Day et al. 1984
	1.01	180	19	21	Eye irritation	Kulle et al. 1987^d^
	2	10	15	53	Eye irritation	Schachter et al. 1986
	2	40	15	60	Eye irritation	Schachter et al. 1987
	3	180	9	Average	Eye, nasal, and throat irritation	Sauder et al. 1986
	3	180	9	Average	Eye, nasal, and throat irritation; FEV_1_ decrease (L)	Sauder et al. 1987
	3.01	20	24	Average	Eye, nasal, and throat irritation	Green et al. 1989^d^
Isophorone	25	15	12	NG	Eye, nasal, and throat irritation	Silverman et al. 1946
Isopropyl acetate	200	15	12	Majority	Eye irritation	Silverman et al. 1946
Isopropanol	400	3–5	10	Majority	Eye, nasal, and throat irritation	Nelson et al. 1943
Methanol	1025	240	1	100	Eye irritation	Apol 1981
Methyl ethyl ketone	100	3–5	10	Majority	Nasal and throat irritation	Nelson et al. 1943^d^
	200	240	19	Average	Subclinical rhinitis	Muttray et al. 2002
Methyl isocyanate	0.5	10	6	100	Eye, nasal, and throat irritation	Smyth et al. 1970
	1.75	1	8	38	Nasal irritation	Smyth et al. 1970
	2	1	4	100	Eye irritation	Kimmerle and Eben 1964
	2.5	120	7	57	Nasal irritation	Pozzani and Carpenter 1963
Nitrogen dioxide	1.5	180	15	Average	Increased airway reactivity (L)	Frampton et al. 1991^d^
	2	60	18	Average	Increased airway reactivity (L)	Mohsenin 1988^d^
	2.5	120	16	Average	Increased airway resistance (L)	Beil and Ulmer 1976
	5	120	16	Average	Increased airway resistance (L)	von Nielding and Wagner 1977
*n*-Pentanol	100	3–5	10	Majority	Throat irritation	Nelson et al. 1943
*n*-Pentyl acetate	100	3–5	10	Majority	Throat irritation	Nelson et al. 1943
Styrene	14.7	15	2	100	Bronchospasm (L)	Moscato et al. 1987
	216	20	3	3	Nasal irritation	Stewart et al. 1968
	600	1	NG	NG	Eye and nasal irritation	Wolf et al. 1956
	800	240	2	100	Eye and throat irritation	Carpenter et al. 1944
Sulfur dioxide	5	300	14	Average	Increase in discomfort, irritation	Andersen et al. 1981
Toluene	100	360	16	Average	Eye irritation	Anderson and Molhave 1983
	100	390	24	Average	Nasal and lower airway irritation	Baelum et al. 1990
	200	210	2	100	Eye and throat irritation	Carpenter et al. 1944
	300	3–5	10	Majority	Eye and throat irritation	Nelson et al. 1943
Toluene-2,4-diisocyanate	0.01	900	15	7	Increased airway resistance (L)	Baur 1985
Triethylamine	4.35	480	2	100	Visual disturbances, discomfort	Akesson et al. 1986
	8.22	240	2	100	Visual disturbances, discomfort	Akesson et al. 1986
	11.6	60	2	100	Visual disturbances, discomfort	Akesson et al. 1986
*p*-Xylene	100	450	11	18	Eye and respiratory irritation	Hake et al. 1981

Abbreviations: FEV_1_, forced expiratory volume in 1 sec; NG, not given. For some studies, multiple experiments were conducted with different exposure times or end points resulting in multiple LOAELs for the compounds.

a Numerical values indicate the percent of subjects responding.

b End points with (L) depict “Lower” respiratory end points; all others are “Upper” respiratory end points.

c ”Average” indicates that the response was a mean response.

d Study was considered to be of higher quality due to study design (e.g., placebo-controlled, blinding, subject selection, subject characteristics, exposure conditions, and/or data reporting).

**Table 2:** RD_50_s of male mice with their corresponding TLVs^a^ and RELs^b^ (ppm), along with the specific strain of mice used in the experiment and reference.

Compound	RD_50_ (ppm)	Exposure time (min)	TLV (ppm)	REL (ppm)	RD_50_ strain	RD_50_ reference
Acetaldehyde	2,845	10	25	NA	SW	Steinhagen and Barrow 1984
	2,932	10	25	NA	B6C3F_1_	Steinhagen and Barrow 1984
	4,946	10	25	NA	SW	Kane et al. 1980
Acetone	23,480	5	500	NA	OF1	de Ceaurriz et al. 1981
	77,156	10	500	NA	SW	Kane et al. 1980
Acrolein	1.03	10	0.1	0.00009	SW	Steinhagen and Barrow 1984
	1.41	10	0.1	0.00009	B6C3F_1_	Steinhagen and Barrow 1984
	1.66	10	0.1	0.00009	BALB/c	Muller and Greff 1984
	1.7	1	0.1	0.00009	SW	Kane and Alarie 1977
	2.9	30	0.1	0.00009	CF1	Nielsen et al. 1984
Allyl alcohol	1.6	5	0.5	NA	OF1	Muller and Greff 1984
	2.5	30	0.5	NA	ICR	James et al. 1987
	3.9	30	0.5	NA	CF1	Nielsen et al. 1984
Ammonia	303	30	25	4.5	SW	Barrow et al. 1978
	789.6	10	25	4.5	CF1	Tomas et al. 1985
*n*-Butyl acetate	730	5	150	NA	OF1	Muller and Greff 1984
*n*-Butanol	1,268	5	20	NA	OF1	de Ceaurriz et al. 1981
	4,784	10	20	NA	SW	Kane et al. 1980
	11,696	30	20	NA	CF1	Kristiansen et al. 1988
Chlorine	3.50	120	0.5	0.07	OF1	Gagnaire et al. 1994
	9.3	10	0.5	0.07	SW	Barrow et al. 1977
	11.97	10	0.5	0.07	BALB/c	Tomas et al. 1985
Ethylacetate	580	5	400	NA	OF1	de Ceaurriz et al. 1981
	614	10	400	NA	SW	Kane et al. 1980
Formaldehyde	3.1	10	0.3	0.076	SW	Kane and Alarie 1977
	4	10	0.3	0.076	BALB/c	Nielsen et al. 1999
	4.9	10	0.3	0.076	B6C3F_1_	Chang et al. 1981
	5.3	5	0.3	0.076	OF1	de Ceaurriz et al. 1981
Isophorone	27.8	5	5	NA	OF1	de Ceaurriz et al. 1981
Isopropyl acetate	4,259	5	100	NA	OF1	Muller and Greff 1984
Isopropanol	5,000	5	200	1.3	OF1	de Ceaurriz et al. 1981
	17,693	10	200	1.3	SW	Kane et al. 1980
Methanol	25,222	5	200	NA	OF1	Muller and Greff 1984
	41,514	10	200	NA	SW	Kane et al. 1980
Methyl ethyl ketone	9,000	10	200	4.5	SW	Stone et al. 1981
	10,745	5	200	4.5	OF1	de Ceaurriz et al. 1981
	31,426	30	200	4.5	CF1	Hansen et al. 1992
Methyl isocyanate	1.3	90	0.02	NA	SW	Ferguson et al. 1986
	2.9	30	0.02	NA	ICR	James et al. 1987
Nitrogen dioxide	349	10	3	0.25	SW	Alarie 1981^c^
Phenol	166		5	1.5	OF1	de Ceaurriz et al. 1981
*n*-Pentanol	4,039	10	NA	NA	SW	Kane et al. 1980
	5,933	5	NA	NA	OF1	Muller and Greff 1984
*n*-Pentyl acetate	1,531	10	50	NA	SW	Alarie 1981a
	1,562	5	50	NA	OF1	Muller and Greff 1984
Styrene	156.3	3	20	5.1	SW	Alarie 1973b
	586	5	20	5.1	OF1	de Ceaurriz et al. 1981
	980	10	20	5.1	SW	Alarie 1981^a^
Sulfur dioxide	117		2	0.25	SW	Alarie et al. 1981^a^
Toluene	3,373	5	50	9.8	OF1	de Ceaurriz et al. 1981
	4,900	10	50	9.8	SW	Dudek et al. 1990
	5,300	30	50	9.8	SW	Nielsen and Alarie 1982
2,4-Toluene	0.24	40	0.005	NA	OF1	de Ceaurriz et al. 1981
Diisocyanate	0.39	30	0.005	NA	SW	Barrow et al. 1978
	0.78	180	0.005	NA	SW	Sangha and Alarie 1979
Triethylamine	156	15	1	0.68	OF1	Gagnaire et al. 1989
	186	30	1	0.68	CF1	Nielsen and Yamagiwa 1989
*p-*Xylene	1,325	5	100	5	OF1	Muller and Greff 1984

NA, not available.

a RELs as described in Collins et al. (2004).

b TLVs developed by ACGIH (2006).

**Table 3:** Summary of linear least-squares regression analyses for various comparisons.

Basic analyses	No. of compounds included	No. of data points included	Regression line	*R*^2^ value
Description of analysis				
All RD_50_s identified in all strains of mice vs. all human LOAELs identified (Figure 1)	25	198	logRD_50_ = 1.16(log LOAEL) + 0.77	0.82
Evaluation using male mice and RELs set by OEHHA for airborne toxicants (Figure 2)	16	37	logRD_50_ = 0.71(log REL) + 2.55	0.71
Evaluation using male mice and the TLV (Figure 3)	24	61	logRD_50_ = 0.86(log TLV) − 1.13	0.86
Addressing issues of human LOAEL variabilities				
Evaluation using all RD_50_s identified in all strains of mice vs. the lowest human LOAEL for each compound	25	58	logRD_50_ = 1.13(log LOAEL) + 1.26	0.81
Analysis for male mice log RD_50_ vs. log LOAEL using lowest RD_50_ values with the lowest LOAEL values	25	25	logRD_50_ = 1.01(log LOAEL) + 1.21	0.77
Analysis for male mice log RD_50_ and human log LOAEL for lower respiratory end points	5	29	logRD_50_ = 1.06(log LOAEL) + 1.21	0.58
Analysis for male mice log RD_50_ and human log LOAEL for upper respiratory end points	23	166	logRD_50_ = 1.22(log LOAEL) + 0.69	0.82
Analysis for male mice log RD_50_ and human log LOAEL for higher quality human studies	7	43	logRD_50_ = 1.40(log LOAEL) + 0.98	0.82
Analysis for male mice log RD_50_ and human log LOAEL for human studies not selected as higher quality	25	155	log RD_50_ = 1.16(log LOAEL) + 0.73	0.79
Evaluating influence of mouse strain				
Evaluation using only Swiss-Webster mice and all human LOAEL values (Figure 2)	19	75	logRD_50_ = 1.12(log LOAEL) + 0.93	0.74
Evaluation using all non–Swiss-Webster mice and all human LOAEL values (Figure 3)	23	120	logRD_50_ = 1.20(log LOAEL) + 0.73	0.83
Evaluating changes in exposure duration				
Evaluation using male mice and human LOAEL values from exposures of ≤ 10 min	16	67	logRD_50_ = 1.27(log LOAEL) + 0.726	0.76
Evaluation using male mice and human LOAEL values from exposures of > 10 min	18	127	logRD_50_ = 1.11(log LOAEL) + 0.838	0.80
Evaluation using male mice and human LOAEL values from exposures of ≥ 60 min	15	101	logRD_50_ = 1.08(log LOAEL) + 0.89	0.80
Log RD_50_ vs. log RD_50_ for RD_50_ values with time < 10 min	16	44	logRD_50_ = 1.04(log LOAEL) + 0.76	0.77
Log LOAEL vs. Log RD_50_ for RD_50_ values with time > 10 min	10	43	logRD_50_ = 1.51(log LOAEL) + 0.56	0.87
Log RD_50_ vs. log LOAEL for RD_50_ values with time equivalent to 10 min	16	111	logRD_50_ = 1.3(log LOAEL) + 0.78	0.80
LogRD_50_ vs. log LOAEL for RD_50_ values at times not equivalent to 10 min	22	86	logRD_50_ = 1.09(log LOAEL) + 0.77	0.8

OEHHA, Office of Environmental Health Hazard Assessment.
